# Integration of Three-Dimensional Scanning and Computer-Aided Design/Computer-Aided Manufacturing (CAD/CAM) Technology in Routine Prosthodontic Practice: A Cross-Sectional Study in Kerala

**DOI:** 10.7759/cureus.76409

**Published:** 2024-12-26

**Authors:** Jamshid Usman M, Neethu Latha, Arya Saraswathy, Hareesh Makkadan Thachanath

**Affiliations:** 1 Prosthodontics, Government Dental College, Kozhikode, IND

**Keywords:** computer-aided design/computer-aided manufacturing, digital dentistry, intraoral scanning, patient satisfaction, prosthodontics

## Abstract

Background: Digital dentistry has transformed all aspects of dentistry, especially prosthodontics, and is increasingly used for diagnosis, treatment planning, execution, student training, and research. This study aimed to assess the perception, attitude, and practice of digital technology in prosthodontics among dental professionals in Kerala, India.

Materials and Methods: A cross-sectional, questionnaire-based study was conducted among dental professionals in Kerala. A self-administered questionnaire with 23 close-ended questions assessed demographics, perception, attitude, and digital technology use in prosthodontics. The questionnaire was distributed via Google Forms (Google LLC, Mountain View, California, United States), and 200 complete responses were analyzed using IBM SPSS Statistics for Windows, Version 25.0 (Released 2017; IBM Corp., Armonk, New York, United States).

Results: The response rate was 81.2%. Among respondents, 60.3% were female and most were aged 31-40 years; 66% were aware of digital technology and 42.5%, primarily males and private practitioners, had used it in practice. Training influenced usage, with 91.3% of non-users lacking training. Intraoral scanning was the most used component (87.2%), and chairside milling was the least used (1.2%). Computer-aided design/computer-aided manufacturing (CAD/CAM) restorations were rated superior to lab-fabricated ones by 75%.

Conclusion: The findings of this study reveal moderate awareness, limited use of digital technology in prosthodontic practice, and significant satisfaction on the part of users with the outcomes. This reinforces the need to develop structured training in digital tools in dental education and offer continuing education for practitioners. A digitally enabled approach to dental education can modernize practices and address existing gaps, enhancing clinical efficiency and patient care through the adoption of digital dentistry.

## Introduction

Digital dentistry has transformed all aspects of dentistry, from diagnosis to treatment planning and impression to prosthesis fabrication. Artificial intelligence for diagnosis, three-dimensional (3D) printing to manufacture precise prostheses and augmented reality in treatment planning are today becoming the catalyst innovations for modern dental practices globally. The advancements increase clinical efficiency, accuracy, and patient outcomes reflecting the global trends in dentistry. The field of dentistry has been revolutionized by digitalization to such an extent that, nowadays, advancement in dentistry has become synonymous with progress in digital dentistry. Digital dentistry incorporates digital and computer-controlled elements like 3D scanning and computer-aided design/computer-aided manufacturing (CAD/CAM) systems to improve accuracy, streamline processes, and enhance patient comfort and quality of dental treatment [1].

No other field of dentistry has been as significantly influenced by digitization as prosthodontics. Prosthodontics uses all fields of digital dentistry, including cone-beam computed tomography (CBCT) systems, intraoral and extraoral scanning, computer-aided design, and additive and subtractive methods for prosthesis fabrication [2]. Initially, digital dentistry was used for crown and bridge fabrication. It has been increasingly used for planning and surgical placement of implants, fabrication of superstructures, and fabrication of removable partial and complete dentures. It has also been found to be immensely useful in patient education, training of dental students, and research [3].

Digital dentistry is changing the way dental practices function, making clinical work more efficient and patient care better. The purpose of this study is to explore the integration of 3D scanning and CAD/CAM technology in routine prosthodontic practice, as it would impact practice and barriers to wider adoption. The benefits of digital dentistry include better patient experience, better diagnostic accuracy, enhanced treatment planning, less chairside time, and fewer appointments [4]. CAD/CAM milling and 3D printing technologies can provide greater fabrication precision with prostheses, leading to an overall better clinical outcome. However, the high initial cost of equipment and lack of training of dental professionals are the challenges. Expansion of the use of digital dentistry in clinical practice is possible through addressing these issues with cost-effective solutions and proper education [5,6].

Nowadays, many dental laboratories are using CAD/CAM systems, computer software, and hardware to design and manufacture products to create precise digital models of dental structures, such as crowns, bridges, and dentures. This is used for prosthesis fabrication because of the increased efficiency and precision [7]. However, complete and efficient utilization of digital technology is possible only if dentists are proficient in it and are using the same in their practice for image acquisition and designing of prostheses. Digital technology can function as an excellent tool for prosthetically driven treatment planning provided the dentist is adept in using CAD.

Studies have been conducted to understand the awareness and use of digital technology in dentistry among dentists and have produced varying results. Studies in Saudi Arabia found that while 99.3% were aware of the use of digital technology [8], only 57% have used chairside CAD/CAM for the fabrication of crowns [9]. Similarly, studies in India determined that 50.8% of dentists have never used CAD/CAM in practice [10], while the awareness regarding the use of digital technology was 98.9% [11]. However, literature on the degree to which digital technology is being utilized by dentists in routine prosthodontic practice, especially in India, seems to be lacking. The component-wise utilization of digital technology, the areas where it is currently being used, and the quality of restorations along with dentist satisfaction will aid in understanding the integration of digital technology in prosthodontic practice and determining the actions needed to facilitate its effective implementation. Hence, this study was undertaken to assess the perception, attitude, and practice of digital technology in prosthodontics among dental professionals in Kerala, India. In particular, the adoption and application of CAD/CAM systems, intraoral scanning, and surgical guides in clinical practice were specifically focused on. The goal of the study was to understand how well practitioners know and are competent with these digital tools. The study also looks at the factors that influence the adoption rates and frequency of use of these technologies in daily prosthodontic procedures. Additionally, it investigates demographic differences in the use of technology, evaluates the perception of digital restorations to traditional methods in terms of perceived quality by clinicians and patients, and reports on the satisfaction of clinicians and patients.

## Materials and methods

A cross-sectional questionnaire-based study was conducted among dental professionals in Kerala, India, between June and August, 2023. Kerala has a unique demographic profile with a large number of private dental practitioners and advanced digital tools in the dental laboratories have established a good network. This was an appropriate setting to investigate what influences the use of digital technology in prosthodontics in a context where healthcare and the dental infrastructure of the region represent the broader trends in India’s changing dental landscape. The study was approved by the Institutional Ethics Committee, Government Dental College, Kozhikode, Kerala, with IEC number 277/2023/DCC (approval number: ECR/673/Inst/KL/2014/RR 20, dated May 18, 2023).

Participants

Dental professionals practicing in Kerala, India, were recruited to participate in this study and complete the self-administered questionnaire on digital technology in prosthodontics. Participants were licensed dental professionals actively engaged in prosthodontic practice including general dentists, prosthodontists, and private practitioners. Only those who gave informed consent and finished the entire questionnaire were included. Responses were excluded if they were incomplete or if the data were inconsistent; participants were excluded if they did not practice in Kerala or did not incorporate prosthodontic procedures in their routine practice. This also resulted in a focused sample of professionals using digital technologies in prosthodontic procedures.

Sample size calculation

The sample size was calculated using the sample size calculator by Raosoft, Inc. (Seattle, Washington, United States) with a 95% confidence interval and a margin of error of 5.52%, which yielded a sample size of 204 respondents. The medium effect (f) was estimated from previous studies on digital technology adoption. The power (1-β error) was set at 0.80, which is recommended for detecting moderate effects in health-related research [12]. Since previous studies conducted in India yielded an 80% response rate, the questionnaire was sent to a total of 250 participants [13].

Data collection

For this study, data were collected from multiple dental clinics and dental colleges across different regions of Kerala, both urban and rural, to represent a diverse range of dental professionals. The regions were Thiruvananthapuram, Kochi, Kozhikode, Thrissur, and Malappuram, as they provided a broader picture of the way digital technology is used in prosthodontics in the state, taking into account the different practices and demography of the patients. Respondents from large cities as well as smaller towns and rural areas were included, representing the range of adoption and usage of digital tools in prosthodontic practice in Kerala. 

Study tool

**Figure 1:**
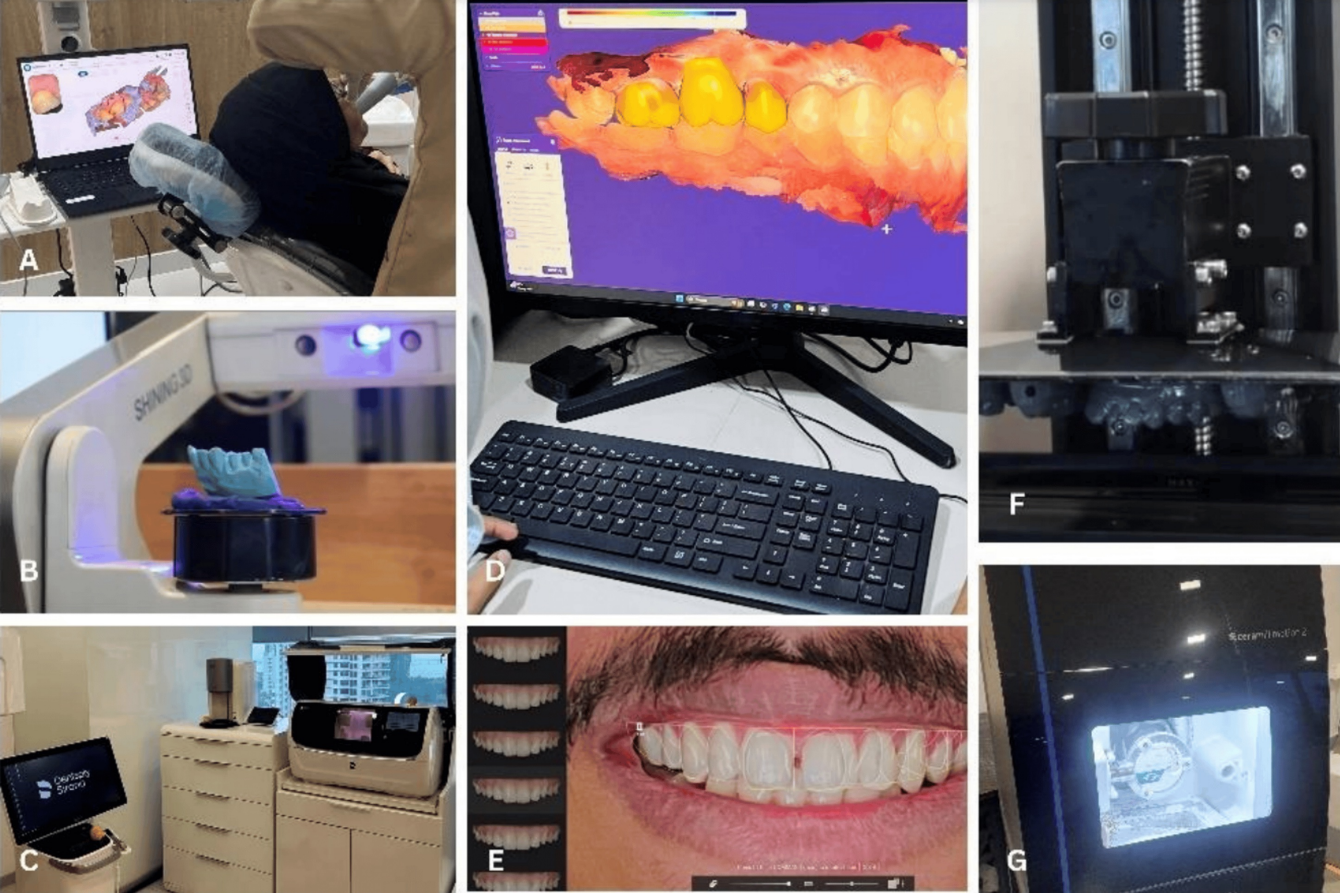
Components of digital dentistry incorporated in routine prosthodontic practice that were asked about in the questionnaire: (A) Intraoral scanning, (B) Laboratory scanning of the cast, (C) Chairside milling unit, (D) Computer-aided designing of fixed dental prosthesis, (E) Digital smile designing, (F) Three-dimensional model printing, (G) Milling of zirconia fixed dental prosthesis Image Sources: Authors Equipment/manufacturers: A. Aoralscan 3 Intraoral Scanner, Shining 3D, Hangzhou, China. B. AccuFab-L4K 3D Printer, Shining 3D, Hangzhou, China. C. CEREC Primemill, Dentsply Sirona Inc., Charlotte, North Carolina, United States. D. exocad GmbH, Darmstadt, Germany. E. Smilecloud SRL, Timisoara, Romania. F. GKtwo 8K resin 3D-printer, UniFormation, Shenzhen, China. G. Ceramill Motion 2, Amann Girrbach, Koblach, Vorarlberg, Austria.

The study instrument was a self-administered questionnaire comprising 23 close-ended questions, divided into four sections (see Appendices). The first section obtained demographic details of the participants, including age, gender, qualification, designation, and clinical experience. The second section assessed perception regarding digital dentistry with six questions, the third assessed practitioners' attitudes towards digital dentistry with five questions, and the fourth section assessed the incorporation of digital technology in prosthodontic practice with seven questions, of which the last five questions could only be accessed by respondents who marked “YES” for the second question of the section on usage of digital technology in practice. The components of digital dentistry incorporated in routine prosthodontic practice which was surveyed using the questionnaire are shown in Figure 1.

A panel of six experts including general dentists, prosthodontists, private practitioners, and teaching faculty assessed content and face validity. Each question was reviewed by the panel to ensure its subject matter, as well as its clarity within the discussion of digital technology use in prosthodontics. The final questionnaire included only questions with a Content Validity Index (CVI) greater than 0.83. For example, the perceived ease of use of CAD/CAM systems and the impacts of digital technologies on the outcomes for patients were elaborated to better characterize how practitioners experienced digital technologies. The panel also suggested rewording the complex items to make them clearer and each question as a faithful reflection of the intended concept. On the basis of the panel's feedback, the questionnaire was refined to improve its robustness and reliability.

The questionnaire was designed in English using Google Forms (Google LLC, Mountain View, California, United States), and the link was distributed to professionals via email and WhatsApp (Meta Platforms, Inc., Menlo Park, California, United States). Personal reminders were sent to participants twice at one-week intervals. A brief description of the study was provided, explaining the purpose and voluntary nature of participation, and informed consent was obtained from participants. To ensure completeness of responses, all questions other than skip patterns were marked as mandatory in the questionnaire. Conditional questions were referred to as skip patterns, which asked only questions appropriate to the respondent based on prior responses. For instance, if a participant said they had never used digital technology in clinical practice, they would skip over questions about particular digital tools. To maintain anonymity, the email IDs of respondents were not collected.

Data analysis

A total of 204 complete questionnaires were received. Statistical analysis was performed using IBM SPSS Statistics for Windows, Version 25.0 (Released 2017; IBM Corp., Armonk, New York, United States). Descriptive statistics were used for frequency distribution and percentages. Associations were examined using a chi-square test and the level of statistical significance was set at p < .05.

## Results

A total of 204 responses were obtained, of which four were incomplete and therefore not included in the analysis. Thus, the response rate for this self-administered questionnaire was 81.2%.

Of the total respondents, 121 (60.3%) were female. Most of the respondents were in the age range of 31-40 years (n = 117) and were graduates (n = 112). Eighty-five respondents (42.5%) had used digital technology for prosthodontic restorations in their clinical practice. Based on this response, the participants were categorized into users and non-users, and an analysis was conducted to understand how these groups differ based on their demographic characteristics, their perception and attitude towards digital dentistry, and whether they had undergone any training in digital dentistry. The demographic characteristics of users and non-users of digital technology in prosthodontic practice are summarized in Table 1.

**Table 1:** Sociodemographic variables and incorporation of digital technology in prosthodontic practice * statistically significant at the 0.05 level

Variables		Number of respondents	Incorporation of digital technology in Prosthodontics		Chi-Square Value	P-value
			Non-user, n (%)	User, n (%)		
Age range	21-30	55	41 (35.7%)	14 (16.5%)	12.54	0.014*
	31-40	117	64 (55.7%)	53 (62.4%)		
	41-50	17	6 (5.2%)	11 (12.9%)		
	51-60	9	3 (2.6%)	6 (7.1%)		
	>60	2	1 (0.9%)	1 (1.2%)		
Gender	Male	79	35 (30.4%)	44 (51.8%)	9.30	0.002*
	Female	121	80 (69.6%)	41 (48.2%)		
Qualification	BDS	112	77 (67.0%)	35 (41.2%)	13.18	0.001*
	MDS	88	38 (33.0%)	50 (58.8%)		
Designation	PG student	9	3 (2.6%)	6 (7.1%)	14.4%	0.006*
	PG students, private practitioners	2	2 (1.7%)	0 (0%)		
	Private practitioners	158	100 (63.29%)	58 (68.2%)		
	Private practitioners , teaching faculty	9	2 (1.7%)	7 (8.2%)		
	Teaching Faculty	22	8 (7.0%)	14 (16.5%)		
Clinical experience	0-5	106	72 (62.6%)	34 (40.0%)	17.52	0.002*
	6-10	48	18 (15.7%)	30 (35.3%)		
	11-15	25	17 (14.8%)	8 (9.4%)		
	16-20	6	3 (2.6%)	3 (3.5%)		
	>20	15	5 (4.3%)	10 (11.8%)		

Statistical analysis revealed that the usage of digital dentistry was significantly higher in male than in female dentists. Across various age groups, the number of users of digital dentistry was significantly higher in the age group of 31-40 years. However, within individual age groups, 41-50 and 51-60 had significantly higher numbers of users than non-users. The use of digital dentistry was statistically significant among postgraduates compared to graduates. When years of clinical experience were compared, a substantial number of users had fewer years of experience, i.e., 0-5 years. Within the various experience-based groups, those with over 20 years of experience had more users than non-users (66.6%), and the 16-20-year group had equal numbers of users and non-users. Postgraduate students and teaching faculty had more users (66.6% and 63.63%, respectively) among them than non-users. Among the groups based on designation, a significant number of users were private practitioners.

When the current perceived knowledge and attitude towards digital dentistry were compared between users and non-users, a considerable number of users reported their knowledge as moderate, and most respondents had a positive attitude towards digital dentistry. The responses to questions assessing the perception and attitude of both users and non-users are shown in Table 2 and Table 3.

**Table 2:** Comparison of participants’ responses to perception-related questions *statistically significant at the 0.05 level.

Questions with responses	Non-user, n (%)	User, n (%)	Chi-Square value	P-value
How do you rate your current perceived knowledge regarding digital dentistry?				
Low	60 (52.2)	6 (7.1)	46.95	0.001*
Moderate	53 (46.1)	71 (83.5)		
High	2 (1.7)	8 (9.4)		
What is your current perceived attitude toward digital dentistry?				
Positive	108 (93.9)	84 (98.8)	3.06	0.07
Not sure	7 (6.1)	1 (1.2)		
What is your current awareness of digital dentistry?				
Yes, aware	62 (53.9)	70 (82.4)	17.69	0.001*
Not aware	5 (4.3)	1 (1.2)		
Not aware but keen to know	48 (41.7)	14 (16.5)		
Do you think digital dentistry will revolutionize prosthodontic work?				
Yes	107 (93)	85 (100)	6.15	0.04*
No	1 (0.9)	0		
Not sure	7 (6.1)	0		
Do you think digital dentistry can be utilized by experts only?				
Yes	17 (14.8)	12 (14.1)	8.50	0.01*
No	71 (61.7)	66 (77.6)		
Not sure	27 (23.5)	7 (8.2)		
Do you think digital dentistry reduces prosthodontic workload?				
Yes	108 (93.9)	81 (95.3)	0.36	0.83
No	1 (0.9)	1 (1.2)		
Not sure	6 (5.2)	3 (3.5)		

**Table 3:** Attitude of users and non-users towards digital dentistry

Questions with responses	Non-user, n (%)	User, n (%)	Chi-Square value		P-value	
Incorporation of digital dentistry into practice will be affordable to patients						
Strongly disagree	4 (3.5)	4 (4.7)		8.62		0.07
Disagree	36 (31.3)	13 (15.3)				
Neutral	49 (42.6)	37 (43.5)				
Agree	23 (20.0)	27 (31.8)				
Strongly agree	3 (2.6)	4 (4.7)				
The use of digital dentistry will reduce patient visit time and the number of appointments						
Strongly disagree	2 (1.7)	0		8.92		0.06
Disagree	2 (1.7)	2 (2.4)				
Neutral	9 (7.8)	5 (5.9)				
Agree	72 (62.6)	40 (47.1)				
Strongly agree	30 (26.1)	38 (44.7)				
The dental curriculum requires modification of digital dentistry						
Strongly disagree	4 (3.5)	0		4.92		0.29
Disagree	0	1 (1.2)				
Neutral	6 (5.2)	4 (4.7)				
Agree	39 (33.9)	34 (40.0)				
Strongly agree	66 (57.4)	46 (54.1)				
How important is training for the use of digital dentistry?						
Extremely important	51 (44.3)	44 (51.8)		4.18		0.24
Very important	43 (37.4)	26 (30.6)				
Important	21 (18.3)	13 (15.3)				
Slightly important	0	2 (2.4)				
How likely are you to dedicate the time and effort to learn digital dentistry and keep pace with this advancing technology?						
Very unlikely	0	1 (1.2)		5.57		0.23
Unlikely	2 (1.7)	0				
Neutral	17 (14.8)	7 (8.2)				
Likely	56 (48.7)	40 (47.1)				
Very likely	40 (34.8)	37 (43.5)				

Training in digital dentistry had a significant effect on the incorporation of digital dentistry in practice. Of the non-users, 91.3% had not undergone any training in digital dentistry. On the other hand, 52.9% of users had undergone some kind of training in digital dentistry (Table 4).

**Table 4:** Users and non-users of digital technology in prosthodontic practice distributed according to training received *statistically significant at the 0.05 level

Training	Non-user, n (%)	User, n (%)	Chi-square value	P-value
No	105 (91.3)	40 (47.1)	47.99	0.001*
Yes	10 (8.7)	45 (52.9)		

**Figure 2:**
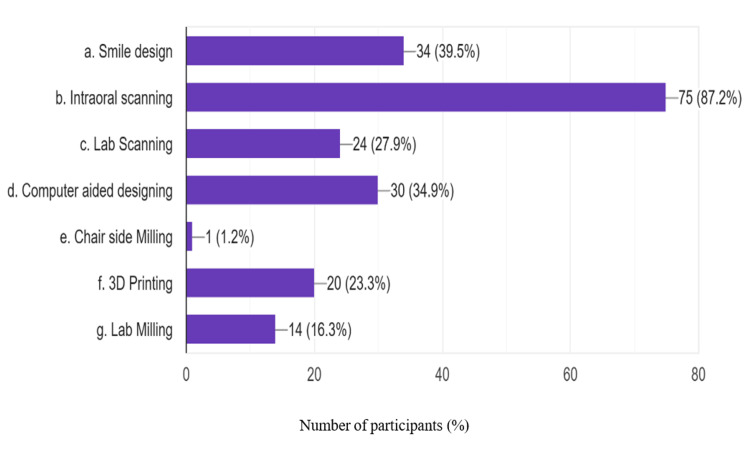
Components of digital dentistry incorporated into prosthodontic practice

Among the components of digital dentistry, most respondents had used intraoral scanning (87.2%), while chairside milling was the least used component (1.2%). The percentage of use of various components of digital dentistry is summarized in Figure 2.

Regarding the prosthodontic procedure for which digital dentistry has been utilized by the respondents, crown and bridge were the most frequent (90.7%), and complete denture was the least (4.7%). The percentage distribution is given inFigure 3.

**Figure 3:**
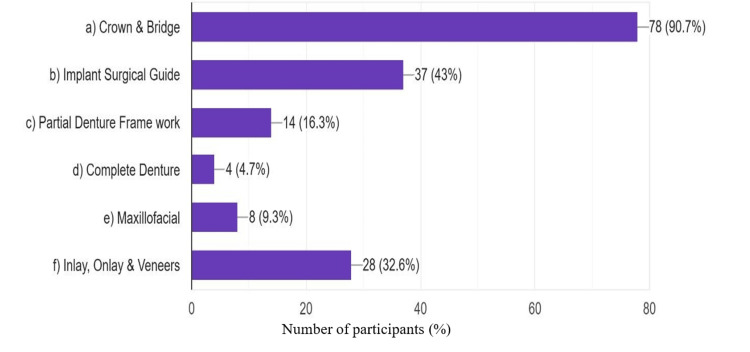
Prosthodontic restoration for which digital technology had been used by the respondents

**Figure 4:**
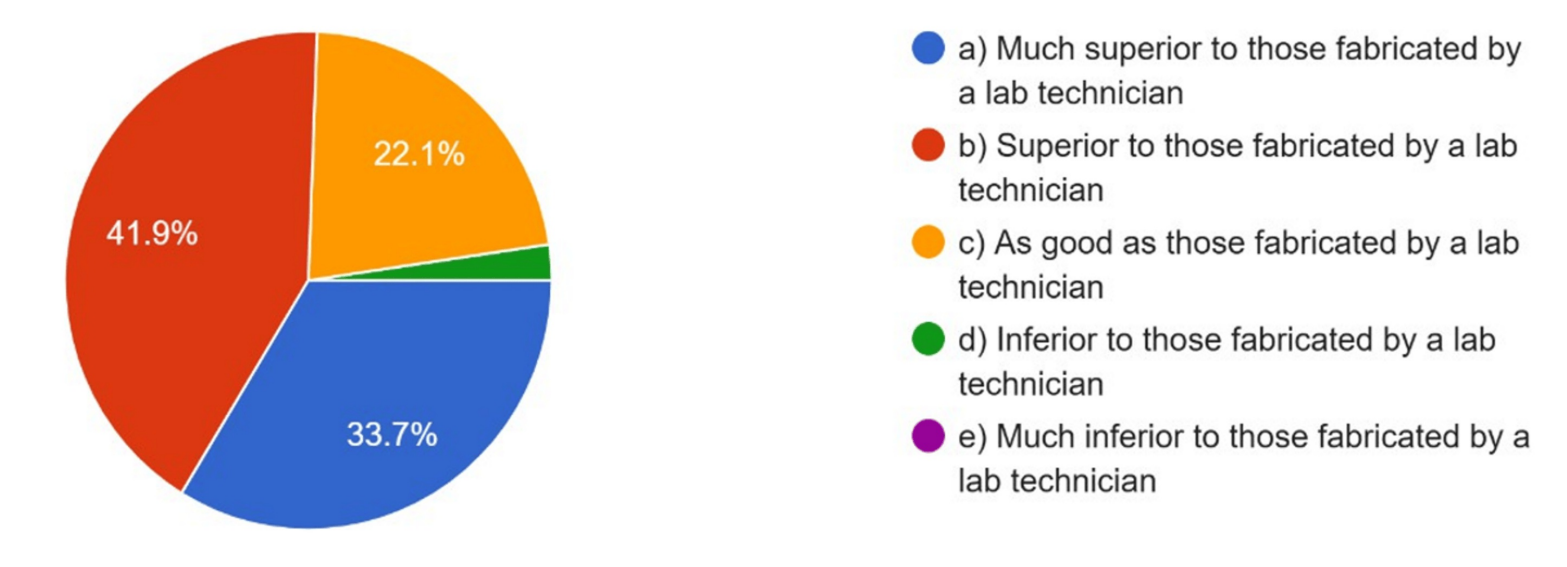
Overall quality of CAD/CAM restorations compared to that fabricated by a lab technician using conventional technique CAD: computer-aided design; CAM: computer-aided manufacturing

**Figure 5:**
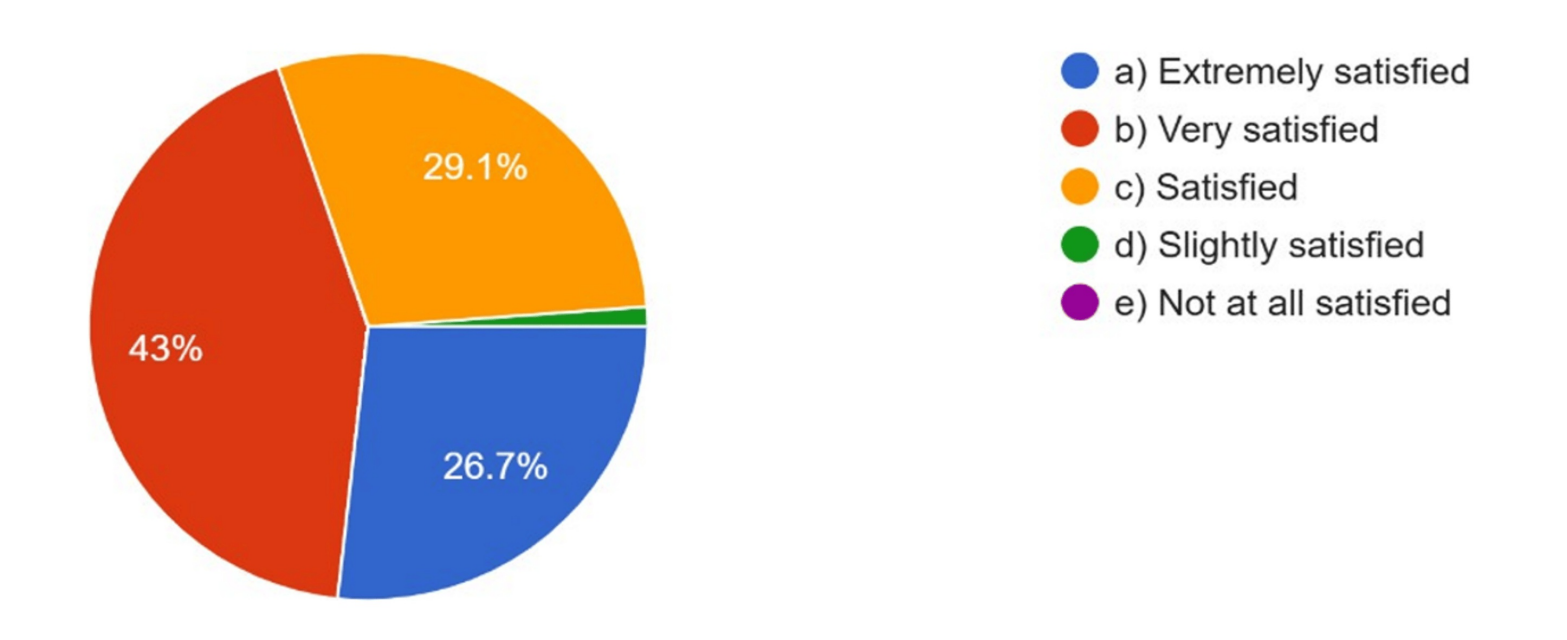
Dentists’ satisfaction with CAD/CAM restorations procedure CAD: computer-aided design; CAM: computer-aided manufacturing

**Figure 6:**
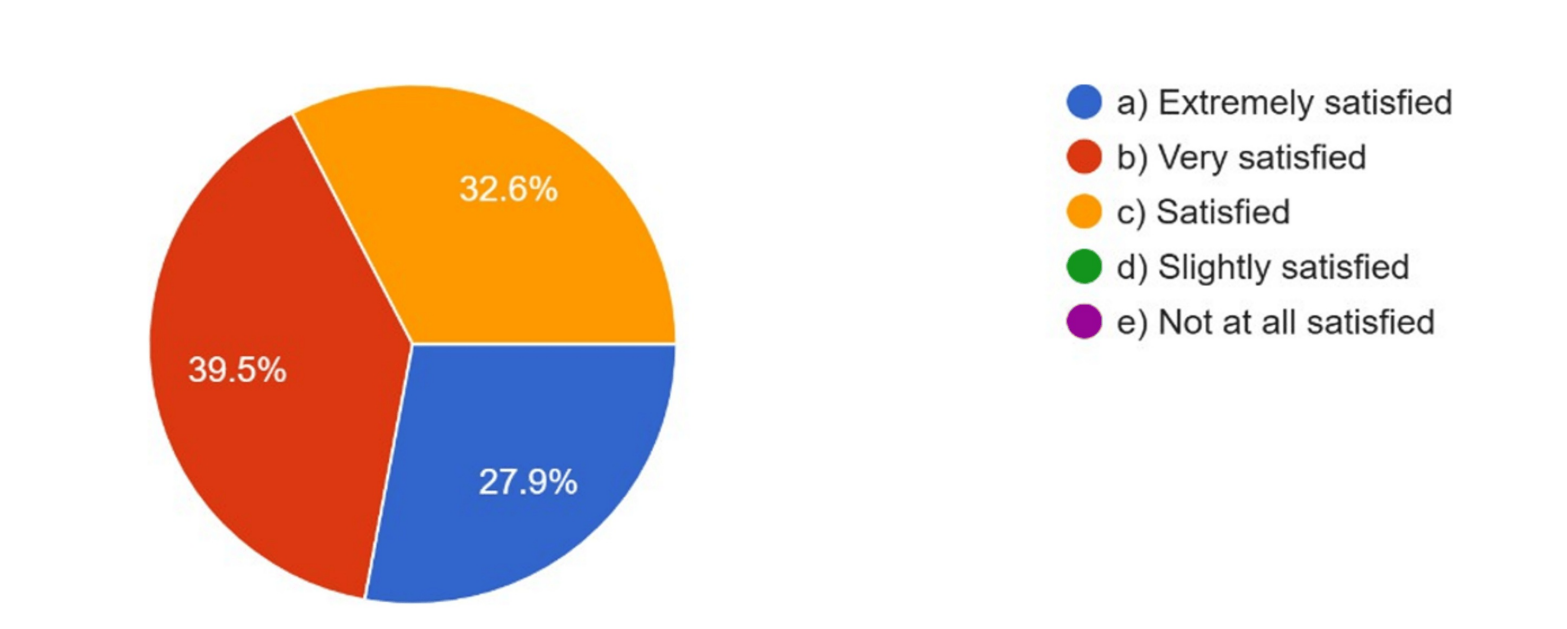
Patients’ satisfaction with CAD/CAM restorations CAD: computer-aided design; CAM: computer-aided manufacturing

The overall quality of CAD/CAM restorations compared to those fabricated by a lab technician, satisfaction with the CAD/CAM restoration procedure, and patients’ satisfaction with CAD/CAM restorations as rated by the respondents are summarized in Figure 4, Figure 5, and Figure 6, respectively.

Around 70.6% of respondents were either extremely or very satisfied with the CAD/CAM procedure, and 75% rated the overall quality of CAD/CAM restorations as much superior or superior to those manufactured by lab technicians. Based on their clinical experience, 68.2% rated patient satisfaction with CAD/CAM restorations as extremely or very satisfying.

## Discussion

Since the first use of CAD-CAM technology recorded in the early 1980s [14], digital dentistry has progressed greatly. With the advent of digital dentistry in this era, it is imperative that we understand the popularity of this among practicing dentists, its use in day-to-day practice, the effect of demographic characteristics and training on its use, and the quality and satisfaction of the patient and clinician with these restorations. The purpose of this study was to evaluate the use of digital technology in routine dental practice and to determine what factors influence its use.

Most of the respondents were below 40 years, female, private practitioners, and had clinical experience between 0 and 10 years. The finding of this study showed that 66% of dentists were aware of digital technology, which is lower than the studies in India conducted by Acharya et al. (98.9%) [11] and Nayaker et al. (96.7%) [15]. Differences in sample composition and regional variations were a possibility for this discrepancy. For instance, the proportion of postgraduate students and specialists in the previous studies was higher than that in this study, which included a greater number of general practitioners with a broader age distribution. Furthermore, studies carried out by Acharya et al. [11] and Nayaker et al. [15] were performed in regions with higher dental care exposure with more advanced dental technologies, which could explain higher awareness levels. The sample for the current study, being balanced for the graduates and post-graduates from both urban and rural areas of Kerala, shows the regional variation in awareness and it is suggested that education and training opportunities in more rural areas have to be enhanced.

Of those who said they did not know about digital technology, 91% said they were willing to learn more, and the majority of respondents said the dental curriculum needs to be changed to focus more on digital dentistry. Digital technologies are being integrated into dental curricula around the world, but integration is vastly different from region to region. Undergraduate education in India still emphasizes classical clinical and laboratory technology with very little exposure to digital tools in prosthodontics [16]. As digital dentistry evolves rapidly, it is important that dental education evolve to keep up with those advancements. Digital technologies should be introduced to the curriculum in a manner that allows for an evolution in the usage of these technologies with workshops and Continuing Dental Education (CDE) programs [17].

Although attitudes and perceptions towards digital dentistry are positive, substitution is not fully realized. Intraoral scanning was the most used component of digital dentistry, used by 87.2% of users, followed by design, and the least used was milling. The lower cost of intraoral scanners compared to full CAD/CAM systems and the practice of dental labs offering scanners to dentists on a case-by-case basis likely explains why intraoral scanners were used more. Furthermore, intraoral scanning also has several advantages to traditional impressions, namely, better efficiency, better patient comfort, and comparable or even better prosthodontic outcomes, and it may be no surprise that intraoral scanning is becoming more popular [18].

The designing of prostheses by 30% of users is encouraging, as it indicates that more and more dentists are becoming involved in the design of prostheses. This can greatly enhance the communication between dentists and laboratories because the design of the prosthesis is in harmony with the dentist's clinical requirements and the patient's needs. Poor communication has traditionally existed between dentists and dental laboratories which has resulted in pontic designs, porcelain types, and prosthesis characterization problems [19,20]. Dentists can avoid many of these common communication issues by designing prostheses directly, which can more clearly convey dentists' expectations. Eventually, this can mean better patient outcomes because the restorations are built to the patient's anatomy and functional requirements.

Crowns and bridges were among the most commonly made prosthodontic restorations manufactured using digital dentistry as were implant treatments, and surgical guides. However, digital tools were the least used for complete denture fabrication. It highlights several challenges in applying digital dentistry to complete dentures, especially the problem of accurately recording soft tissue and maxillomandibular relations. This is similar to the findings of Alnafaiy et al., who assessed digital technology implementation in Saudi Arabian dental institutions and found that digital technology was most used in the fabrication of single crowns and implant-supported crowns and least for complete dentures [21]. Digital denture fabrication has progressed much later than crowns, with important innovations made by pioneers such as Goodacre, who developed a prototype for digital denture construction [14]. The potential of digital dentures is promising; however, the higher costs of laboratory equipment and the requirement of system-specific training are challenges. In addition, recording soft tissues and determining lip support is difficult and may limit widespread adoption in routine practice [22]. Yet, with the development of materials, techniques, and systems, digital dentures could become more popular, particularly for their potential to lower patient appointment times and in-office printing.

In this study, respondents rated the overall quality of CAD/CAM restorations as being superior to restorations fabricated by lab technicians, with 74.6% of respondents rating the overall quality of restorations superior to lab technician restorations, and 67.4% indicating high patient satisfaction with CAD/CAM restorations. These findings are consistent with previous research, for instance, that of Nassani et al., which also found similar amounts of satisfaction and quality [9]. High satisfaction rates suggest digital technology is achieving better quality prosthodontic restorations and better patient outcomes.

Strengths and limitations of the study

A strength of this study is that it included a broad geographic scope of Kerala, a state with the third largest number of registered dentists and a high reliance on dental labs that use state-of-the-art technology. It provides valuable information on the factors and challenges of integrating digital technology in dental practice in India. Additionally, studies that examine the use of digital technology by users in South India are scarce, which makes this study an important contribution to our understanding of how digital tools are being used in prosthodontic practice in this region.

One major limitation of the study is that most respondents were under 40 years of age, which means they were in groups with less than 10 years of clinical experience. This could also lead to sample bias because we can't assess the use of digital technology amongst practitioners who have more than 10 years of practice, especially those who are aged 40 years and older. The study also did not investigate what prevents some practitioners, especially non-users, from integrating digital dentistry into their practice. Understanding these barriers is an important area for future research because this can suggest how to organize targeted interventions and training programs. Future studies will benefit from a greater breadth of age and greater diversity of experience level with respect to clinical entrustment for the generation of understanding about the integration of digital dentistry at different levels of professional development. Furthermore, research into the issues encountered by non-users and the barriers to adoption will help reveal the particular educational and technical gaps that must be closed in order to broaden the acceptance and use of digital tools in prosthodontics.

## Conclusions

Digital technology awareness in prosthodontic practice among surveyed dentists is moderate due to a lack of proper training that significantly impacts its adoption. Mandatory digital dentistry modules can be integrated into dental curricula in order to improve this, including technologies in the field of CAD/CAM and intraoral scanning. Practical, hands-on training with industry leaders could be a collaboration that could help students learn to use these tools in clinical practice. The most commonly fabricated restorations using digital technology are crowns and bridges, and the most popular digital component is intraoral scanning. CAD/CAM restorations were much liked by dentists, especially in comparison to traditional methods. This finding suggests that digital technology investment could increase treatment quality and patient satisfaction, and thus enhance the case for digital tools as a standard of care for policymakers and dental practices. These results point to the need for further work to explore the barriers to adoption, in particular in underserved areas, and to determine the long-term effects of digital dentistry on clinical outcomes and patient care. Further exploration of these areas will facilitate broader integration of digital technologies into prosthodontic practice.

## Figures and Tables

**Figure 7:**
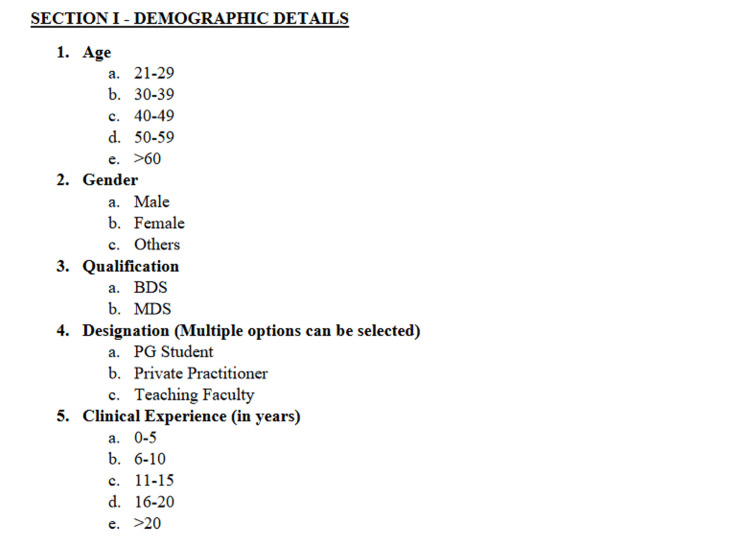
Section 1

**Figure 8:**
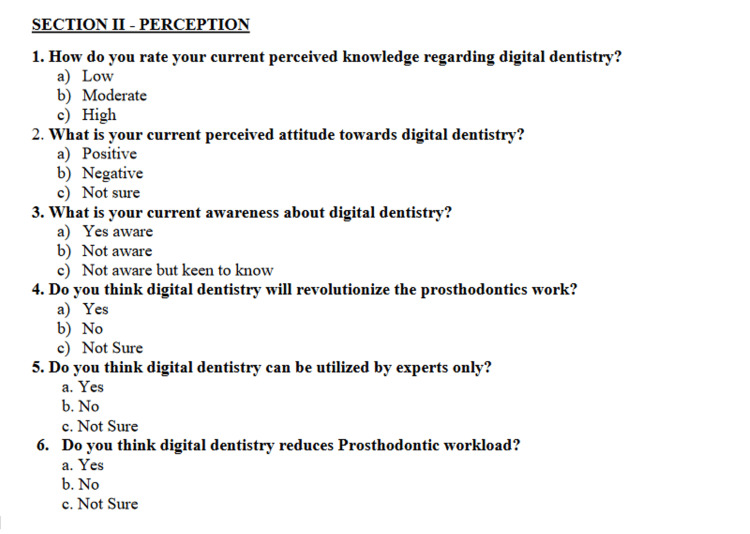
Section 2

**Figure 9:**
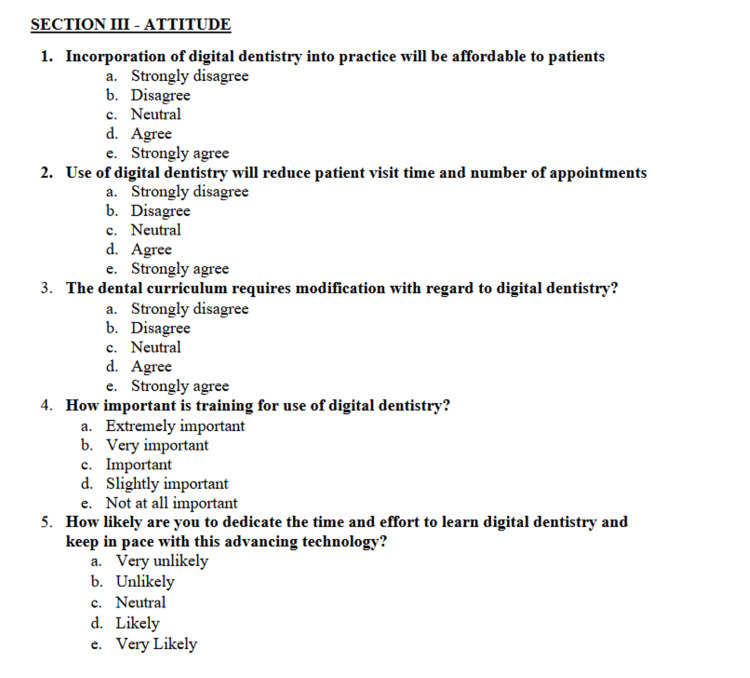
Section 3

**Figure 10:**
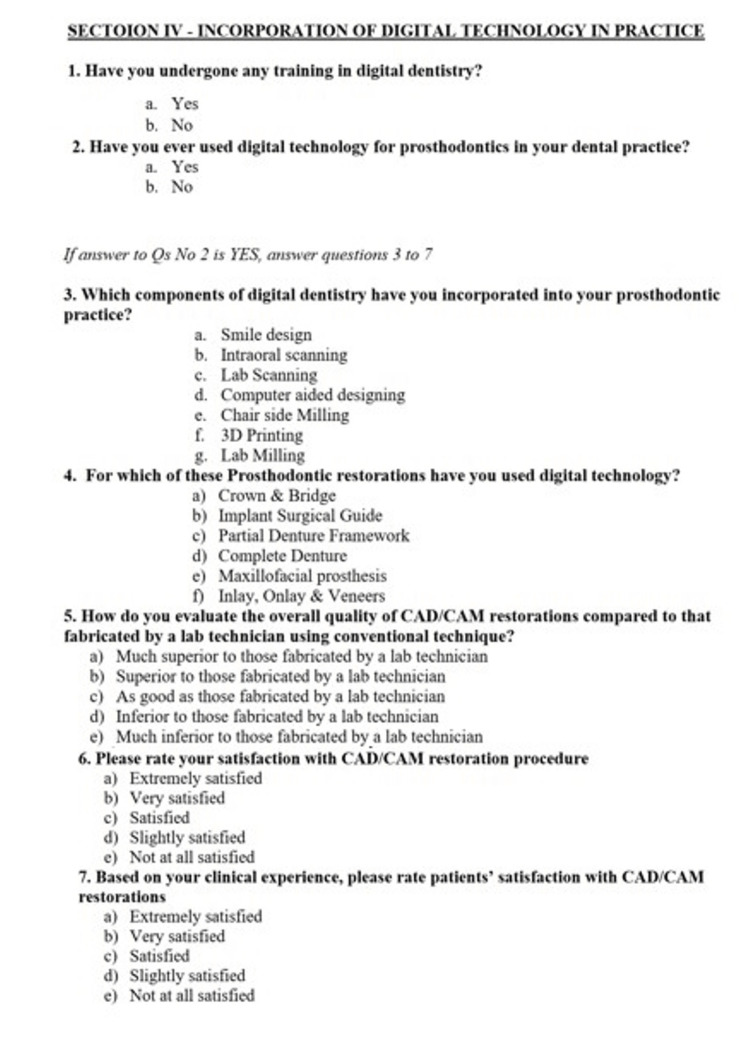
Section 4

## Appendices

Questionnaire
